# Investigation of Andrographolide Effect on Non-Infected Red Blood Cells Using the ^1^H-NMR-Based Metabolomics Approach

**DOI:** 10.3390/metabo11080486

**Published:** 2021-07-28

**Authors:** Ashraf Ahmad Issa Alapid, Roslaini Abd. Majid, Zaid O. Ibraheem, Ahmed Mediani, Intan Safinar Ismail, Ngah Zasmy Unyah, Sharif Alhassan Abdullahi, Norshariza Nordin, Mohammed Nasiru Wana, Rusliza Basir

**Affiliations:** 1 Medical Parasitology Unit, Department of Medical Microbiology and Parasitology, Faculty of Medicine and Health Sciences, Universiti Putra Malaysia, Serdang 43400, Selangor, Malaysia; asalapid82@gmail.com (A.A.I.A.); ngah@upm.edu.my (N.Z.U.); 2 Department of Zoology, Faculty of Science-Alasaba, University of Gharyan, Gharyan 010101, Libya; 3 Faculty of Medicine and Defence Health, National Defence University of Malaysia, Kuala Lumpur 57000, Malaysia; roslaini@upnm.edu.my; 4 Department of Pharmacy, Al Rafidain University College, Al Mustansyria, Baghdad 10052, Iraq; zaid.2002.205@gmail.com; 5 Institute of Systems Biology (INBIOSIS), Universiti Kebangsaan Malaysia, Bangi 43600, Selangor, Malaysia; medianiahmed47@gmail.com; 6 Natural Medicine and Products Research Laboratory, Institute of Bioscience, Universiti Putra Malaysia, Serdang 43400, Selangor, Malaysia; safinar@upm.edu.my; 7 Department of Medical Microbiology and Parasitology, Faculty of Clinical Sciences, Bayero University Kano, Kano 700241, Nigeria; asabdullahi.mcp@buk.edu.ng; 8 Department of Biomedical Sciences, Faculty of Medicine and Health Sciences, Universiti Putra Malaysia, Serdang 43400, Selangor, Malaysia; shariza@upm.edu.my; 9 Department of Biological Sciences, Faculty of Science, Abubakar Tafawa Balewa University Bauchi, Bauchi 740272, Nigeria; mwnasiru@atbu.edu.ng; 10 Pharmacology Unit, Department of Human Anatomy, Faculty of Medicine and Health Sciences, Universiti Putra Malaysia, Serdang 43400, Selangor, Malaysia

**Keywords:** andrographolide, chloroquine, uninfected RBCs, ^1^H-NMR, metabolic pathway, anti-malarial

## Abstract

Andrographolide (AG) has been shown to have several medicinal and pharmaceutical effects, such as antimicrobial, anti-inflammatory, antioxidant, anti-diabetic, and anti-malarial activities. Moreover, studies to assess the pharmacological effect of AG on the metabolic changes of uninfected red blood cells (uRBCs) have not yet been investigated. This study aims to evaluate the pharmacological effects of AG compared to chloroquine (CQ) on the metabolic variations of uRBCs in vitro using a proton nuclear magnetic resonance (^1^H-NMR)-based metabolomics approach coupled with multivariate data analysis (MVDA). Forty-one metabolites were successfully identified by ^1^H-NMR. The results of the unsupervised data analysis principal component analysis (PCA) showed ideal differentiation between AG and CQ. PC1 and PC2 accounted for 71.4% and 17.7% of the explained variation, respectively, with a total variance of 89.10%. Based on S-plot and VIP values, a total of 28 and 32 metabolites were identified as biomarkers in uRBCs-AG and uRBCs-CQ, respectively. In uRBCs treated with AG, ten metabolic pathways were determined to be disturbed, including riboflavin metabolism, d-glutamate and d-glutamine metabolism, phenylalanine metabolism, glutathione metabolism, proline and arginine metabolism, arginine biosynthesis, citrate cycle, glycolysis/gluconeogenesis, and pyruvate metabolism as well as alanine, aspartate, and glutamate metabolism. In contrast, in CQ-treated uRBCs, nine affected metabolic pathways were determined, which involved the same metabolic pathways for uRBCs-AG, except for glutathione metabolism. These findings suggest an evident relationship between AG and CQ associated with metabolic changes in intact RBCs after being exposed to the treatment. The metabolomics results could allow useful comprehensive insights into the underlying mechanism of the action of AG and CQ on red blood cells. Consequently, the ^1^H-NMR-based metabolomics approach was successfully utilized to identify the pharmacological effects of AG and CQ on the metabolic variations of uRBCs.

## 1. Introduction

Human red blood cells (RBCs), also known as erythrocytes, represent a high number of types of blood cells in the human body. They also account for nearly half of the total blood volume and their diameters are 7–8 µm [1]. Normal human RBCs are composed of high concentrations of different metabolites that act as coenzymes of redox reactions, such as nicotinamide adenine dinucleotide phosphate (NADP^+^ and NADPH) and oxidized/reduced nicotinamide adenine dinucleotide (NAD^+^ and NADH). In addition, they contain enzymatic antioxidants, such as catalase (Cat), superoxide dismutase (SOD), glutathione peroxidase (GPx), and peroxiredoxin 2 (Prx2). The coenzymes of energy are also present, such as adenosine diphosphate (ADP), adenosine monophosphate (AMP), and adenosine triphosphate (ATP), as well as non-enzymatic antioxidants, such as oxidized and reduced glutathione (GSSG and GSH). All of these components can be found in plasma/serum [2,3].

Unlike several other cells in the human body, mature RBCs lack nuclei, mitochondria, ribosomes, and other organelles, which are unable to generate energy through the Krebs cycle, and synthesize new proteins [4,5]. For this reason, the anaerobic degradation of glucose via the glycolytic pathway known as the Embden–Meyerhof pathway (EMP) is the only source of produced ATP and NADH [6]. It is very important to note that RBCs are highly metabolically active cells and glycolysis is the main metabolic pathway in RBCs that provides the energy demand. They also have a function to protect cells against reactive oxygen species (ROS) using the GSH antioxidant [2]. RBCs, as the most abundant types of blood cells, work as a perfect candidate for in vivo drug delivery due to many characteristics, including an extended circulation period of ~120 days, flexibility, superb biocompatibility, and minimal immunogenicity [7,8,9,10]. Due to these remarkable properties, RBCs have been widely studied as drug delivery systems [11].

Metabolomics can study the profiling of the biochemical compounds or metabolites which are changed by the physiological or pathological state in cells, tissue, biofluids, organs, and organisms [12,13]. Many analytical tools have been employed in metabolomics studies, of which proton nuclear magnetic resonance (^1^H-NMR) spectroscopy is the major one. Its significant role is in the analysis of the metabolites in different biological samples, including RBCs, serum, plasma, and urine [14]. Yet, blood analysis using metabolomics is limited to the analysis of either serum or plasma metabolites only, while all other blood components obtained during sample preparation, including the RBCs, white blood cells (WBCs), and platelets (PLT) are barely examined [2]. So far, only a handful of researchers have studied the metabolomics variation of RBCs and infected RBCs with different species of *Plasmodium* parasites using ^1^H-NMR coupled with multivariate data analysis (MVDA) [2,15,16,17]. In addition, metabolomics can be used to evaluate the changes in metabolites of blood cells upon treatment with herbal drugs.

*Andrographis paniculate* is one of the medical plants which has been used extensively in Southern Asia, China, and India for many clinical applications [18]. *A. paniculate* has many bioactive compounds, one of which is andrographolide (AG). It is a labdane diterpenoid derivative reported to exhibit many medicinal and pharmaceutical attributes, such as antimicrobial, anti-inflammation, antioxidant [19], cardio-protection, hepatoprotection [20], anti-HIV, anti-carcinogenic [21,22], anti-diabetic, anti-trypanosomal activity [23], and anti-malarial [24,25,26,27,28,29,30,31]. Concerning the effect of AG as an anti-malarial drug, there is one study that has reported the impact of AG on the membranes of uninfected RBCs (uRBCs) and infected RBCs (iRBCs) by *Plasmodium falciparum* 3D7. The results showed that AG was harmless to uRBCs at concentrations approach in its medicinal effect on *Plasmodia*. However, this stability declined at higher concentrations [30]. In iRBCs, a high concentration of AG inhibits the *Plasmodium*-induced permeation pathway as well as the capability of merozoites to infect new RBCs [30]. In this context, AG has many pharmacological activities, when used as an anti-malarial drug on both uRBCs and iRBCs with the *Plasmodium* parasite. More significantly, mature RBCs do not have any genetic elements, making them less risky compared to other cell therapies and genes [5]. Undoubtedly, AG may have an effect on the metabolism of uRBCs and iRBCs. However, there is no study of the AG effects on the metabolism of uRBCs of the host, especially when using it as an anti-malarial drug. Nevertheless, many studies suggested that RBCs might contribute to the metabolism of exogenous and endogenous elements, including drugs [32,33]. Consequently, understanding the impact of these elements or drugs on intact RBC metabolism and its biology may have critical outcomes in the field of antimalarial drug discovery. Metabolites of RBC as the downstream of gene expression can show the real changes upon treatment with drugs. Metabolites have numerous activities in cell physiology, including fuel, structure, signaling, stimulating, and inhibiting enzyme effects; catalytic activity as an enzyme cofactor, defense, and interactions with others. To the best of our knowledge, this study was carried out to fill up this gap. In addition, there has been no study conducted to investigate and interpret the metabolic changes associated with AG on uRBCs. Therefore, this study aimed to evaluate the metabolic variations of uRBCs following exposure to AG and compared with chloroquine (CQ) by using a ^1^H-NMR-based metabolomics approach. It is essential to assess the effects of AG on uRBCs in vitro when intending to reveal some potent insights into the field of antimalarial drugs with a novel mechanism of action.

## 2. Results and Discussion

### 2.1. ^1^*H-NMR Spectra of Uninfected RBC Extracts*

**Figure 1:**
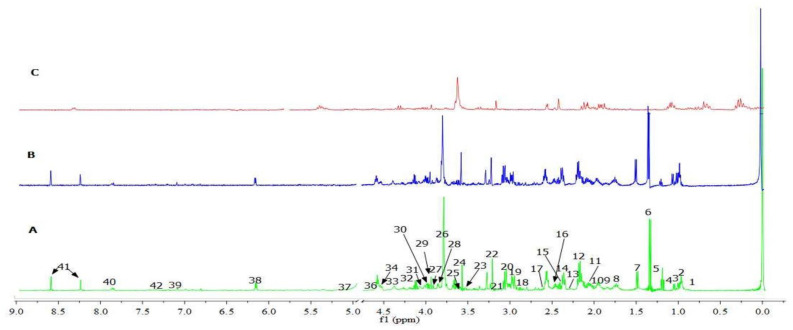
^1^H-NMR spectra (500 MHz, CPMG) of untreated uRBCs-control (**A**), uRBCs-AG (**B**), and uRBCs-CQ (**C**). The ^1^H-NMR signals identified were: 1; isovalerate, 2; 2-aminobutyric acid, 3; valine, 4; isobutyrate, 5; 3-hydroxybutyrate, 6; la-tate, 7; alanine, 8; lysine, 9; ornithine, 10; proline, 11; homoserine, 12; methionine, 13; acetone, 14; glutamate, 15; pyruvate, 16; l-glutamine, 17; riboflavin, 18; citrate, 19; glutathione, 20; creatine, 21; cis-aconitic acid, 22; choline, 23; glucose, 24; glycine, 25; threonine, 26; arginine, 27; betaine, 28; ribose, 29; mannose, 30; fructose, 31; isocitrate, 32; pyroglutamic acid, 33; n-acetylcysteine, 34; 2-phosphoglycerate, 35; guanosine triphosphate (GTP), 36; 4-pyridoxate, 37; adenosine triphosphate (ATP), 38; histidine, 39; 3-methylhistidine, 40; adenosine monophosphate (AMP), 41; phenylalanine.

The representative 500 MHz ^1^H-NMR spectra of the extracts of uRBCs-untreated, uRBCs-AG and uRBCs-CQ are shown in Figure 1. By examining the spectra of the three samples, there were variations in the intensity of the peak signals, wherein the uRBCs-AG revealed a lower intensity compared to the those of uRBCs-CQ as well as uRBCs-control (Figure 1). The metabolite signals were then assigned based on the previous studies [2,34], Human Metabolome Database (HMDB), and the library of Chenomx NMR suite 7.5 (Chenomx Inc., Canada) by comparison with the ^1^H-NMR signals of reference compounds. Human red blood cells contain a wealth of metabolites, such as amino acids, carbohydrates, fatty acids, and coenzymes. In addition, RBCs represent a high percentage of total blood cells [2,34]. In this study, 41 blood metabolites were identified as shown in Table 1. All these metabolites have been previously reported in many studies [16,17,35,36]. The metabolites belonged to different chemical classes, such as fatty acids, amino acids, organic acids, and carbohydrates. The first region of the spectra from 0.9 to 3.5 ppm exhibited signals that primarily belong to fatty acids (i.e., isovalerate and 3-hydroxybutyric) as well as amino acids (i.e., valine, lysine, alanine, arginine, threonine, and glutamine). The middle region of the spectra (3.5–5.6 ppm) showed the signals of carbohydrates (i.e., fructose, glucose, and ribose) and some amino acid signals (i.e., glycine, threonine, arginine. The remaining part of the spectra (5.7–9.0 ppm) showcased signals of organic acids and purine nucleotides (i.e., lactate, isocitric acid, adenosine monophosphate (AMP), and adenosine triphosphate (ATP). Table 1 summarizes all the identified metabolites of uninfected RBC extracts, and their characteristic signals.

### 2.2. Principal Component Analysis (PCA) of ^1^H-NMR Data

**Figure 2:**
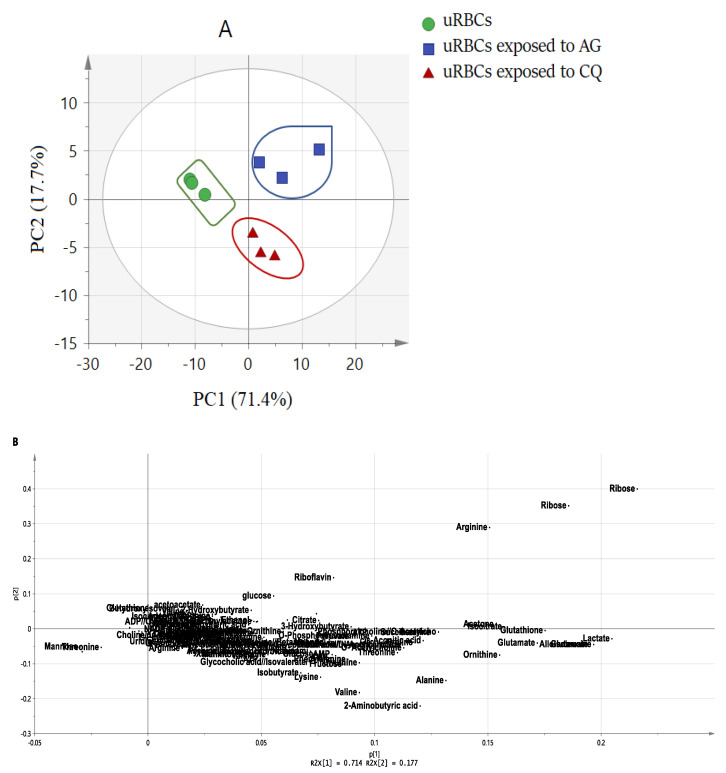
Principal component analysis (PCA) of the uninfected red blood cells’ metabolic profiles: (**A**) score scatter plot (PC1 vs. PC2), (**B**) the loading scatter plot, uRBCs; uninfected red blood cells, AG; andrographolide, CQ; chloroquine.

Principal component analysis (PCA) is one of the most practical and reliable MVDA methods. As an unsupervised method, PCA is employed to minimize the dimensionality of the multivariate dataset. PCA is generally presented graphically as a score and loading plots that are used to determine the similarities or the metabolic differences between biological samples. Therefore, one of the benefits of PCA is that it can help in showing the occurrence of clusters or outliers between the samples represented in the score plot. In order to determine the metabolites that have contributed to a separation in the score plot, the loading plot is used. This plot shows the variables that are responsible for the discernment pattern between the samples as well as the recognition of spectral signals resulting from the discovery of the compounds [37]. The binned ^1^H-NMR data were subjected to PCA in order to distinguish the variations or similarities of the blood metabolites among the treated and untreated uninfected RBCs samples. The PCA model showed a clear separation between three groups into three clusters (Figure 2A). The model has great goodness of fit, with R2Y = 0.968, and good predictability, with Q2 = 0.906. In the PCA score plot, the first two principal components, PC1 and PC2, revealed a total variance of 89.10%, with PC1 accounting for 71.4% and PC2 for 17.4%. The control group was separately discriminated from the other two treated groups with AG and CQ by PC1. The uRBCsCQ group was separated from the control and uRBCsAG by PC2 (Figure 2A). No outlier data were observed in the X-space based on the DModX analysis [38].

The loading scatter plot was performed in order to know the characteristic variables that contributed to the separation in score plot. Consequently, both the loading and score plots are complementary to each other and aid the comprehensive insight into the differentiation between the untreated uRBCs, uRBCs-AG and uRBCs-CQ (Figure 2B). The uRBCs were located on the negative side of PC1, characterized as having lower level/concentration of most blood metabolites, while the two uRBCs treated groups (uRBCs-AG and uRBCs-CQ) were on the positive side of PC1 with higher levels of blood metabolites. The metabolite signals in the upper-right quadrant of the scatter plot contribute to the separation of uRBCs-AG, including ribose, arginine, glucose, acetone, citrate, 4-pyridoxate, 3-hydroxybutyrate, and riboflavin. On the other hand, in the uRBCs-CQ group, the contributing metabolite signals were in the lower-right quadrant of the scatter plot belonging to the metabolites, such as 2-aminobutyric acid, valine, isobutyrate, lactate, alanine, lysine, glutathione, and glutamate. The results of the loading scatter plot indicate that the metabolites of the uRBCs-CQ group were higher compared to uRBCs-AG (Figure 2B). The observed higher level of metabolites in both treated groups could be attributed to AG and CQ. Both AG and CQ showed significant effects by shifting away from the untreated group. A possible explanation of these effects or changes in the normal components of RBCs might be the immediate result of an established pharmacologic action of this drug [39]. In addition, AG was possibly innocent to RBCs at concentrations of its therapeutic level against plasmodia, which can be reduced at higher concentrations [30]. To find the biomarkers for both AG and CQ, separate models for each group of uRBCs-AG and uRBCs-CQ were constructed by comparing them with the uRBCs control group.

### 2.3. Biomarker Identification by OPLS-DA

**Figure 3:**
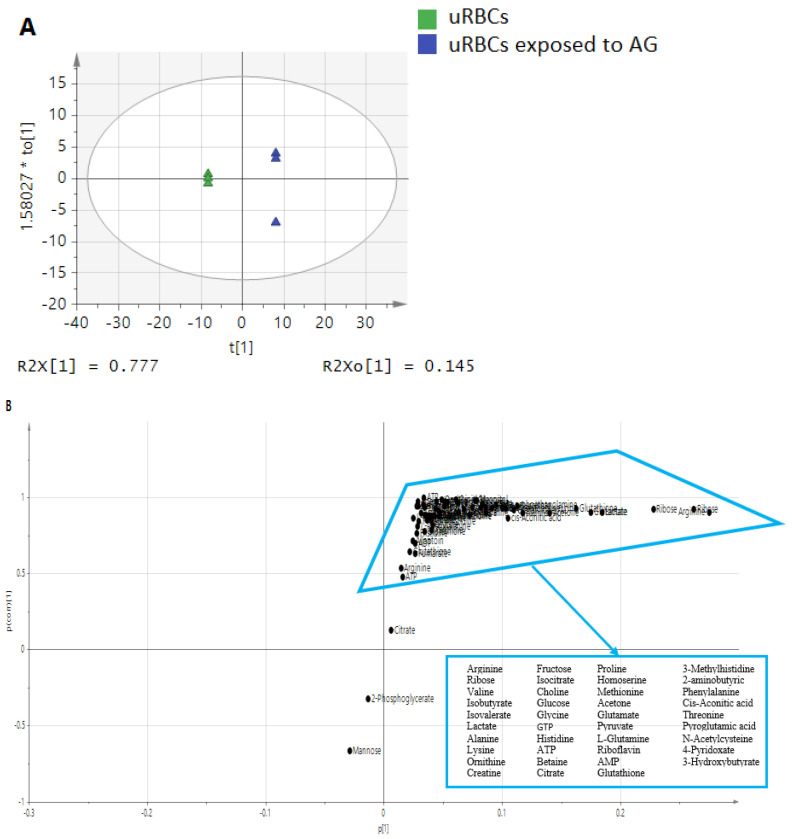
The OPLS-DA score scatter plot (**A**) and S-plot (**B**) of the blood metabolic profiles of uninfected RBC samples. uRBCs; uninfected red blood cells, AG; andrographolide.

In most of the metabolomics studies conducted to determine biomarkers, OPLS-DA is the preferred method and has proven to be a very helpful tool for this purpose. In the OPLS-DA score plot, S-plot and variable importance in projection (VIP) values help in assigning the significant metabolites, which contribute to the biological outcomes [40]. Generally, variables with VIP values equal to or higher than 1.0 are considered to be significant features in the OPLS-DA model [38]. In this study, the OPLS-DA was employed on blood ^1^H-NMR data to reveal distinct observations about the significant metabolites in the models of uRBCs-AG versus uRBCs and uRBCs-CQ versus uRBCs. The score plot of OPLS-DA revealed an obvious separation by PC1 between uRBCs and uRBCs-AG. The uRBCs group was on the positive side of PC1, while the group treated with AG was on the negative side (Figure 3A).

The S-plot is the covariance and association loading diagnostics of the OPLS-DA model, which gives an overview of the affecting variables on the model and clarifies significant metabolites in the projection (Figure 3B). It shows that there were significantly higher metabolites in the uRBCs treated with AG, such as arginine, ribose, cis-aconitic acid, lactate, alanine, isovalerate, valine, acetone, glutamate, and riboflavin. These metabolites contributed to the separation in the upper-right quadrant of the S-plot, which have the covariances and positive correlations with uRBCs-AG. However, the other metabolites, such as mannose and 2-phosphoglycerta, were in the lower-left quadrant, which were higher in uRBCs, as illustrated in Figure 3B. The results indicate that these metabolites were associated with AG’s effect, and they might be the biomarkers.

**Figure 4:**
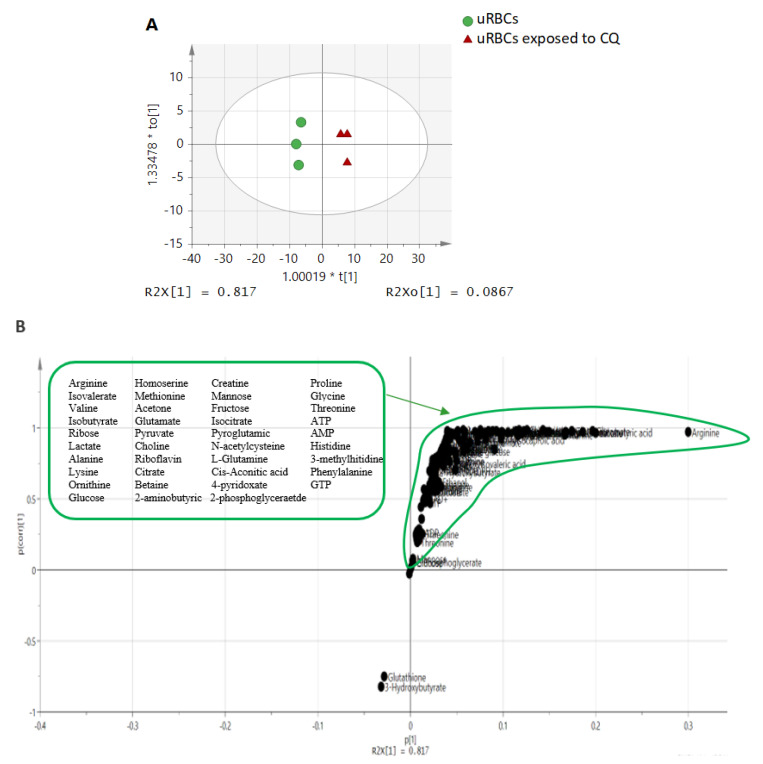
The OPLS-DA score scatter plot (**A**) and S-plot (**B**) of the blood metabolic profiles of uninfected RBC samples. uRBCs; uninfected red blood cells, CQ; chloroquine.

In the other OPLS-DA model, an obvious distinction by PC1 between uRBCs and uRBCs-CQ was shown in the OPLS-DA score plot with great discriminant statistical values of R2 and Q2 represented as 0.903 and 0.934, respectively. The uRBCs group was on the left side of component 1, while uRBCs treated with CQ were on the right side of PC1 (Figure 4A). These results are consistent with those previously found in the PCA model. Figure 4B represents the S-plot. There are substantially higher metabolites in the group of uRBCs treated with CQ, such as arginine, lactate, glutamate, 2-aminobutyric, alanine, valine, isocitrate, acetone, threonine, proline, creatine, and lysine, which contribute to separation in the upper right side of the S-plot. At the same time, the bottom-left of the plot shows the metabolites which were low, such as 3-hydroxybutyrate and glutathione. Based on these findings, these metabolites are significantly affected by CQ and are possibly the biomarkers.

**Figure 5:**
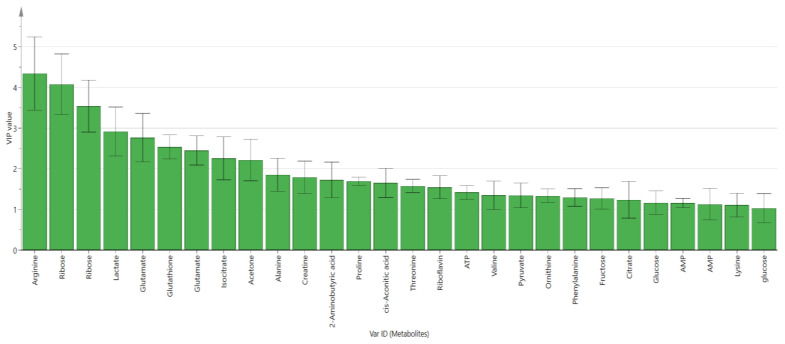
Variable importance of projection (VIP) values of the influential contributing metabolites in the OPLS-DA score plot of uninfected RBCs-control and uRBCs-CQ, error bars represent the standard error of the mean (SEM), uRBCs; uninfected red blood cells, AG; andrographolide.

The variable importance of projection (VIP) showed that 28 metabolites were identified as biomarkers from the OPLS-DA model of the uRBCs treated with AG. The VIP > 1.0 metabolites were arginine, ribose, ribose, lactate, glutamate, glutathione, glutamate, isocitrate, acetone, alanine, creatine, 2-aminobutyric acid, proline, cis-aconitic acid, threonine, riboflavin, ATP, valine, pyruvate, ornithine, phenylalanine, fructose, citrate, glucose, AMP, AMP, lysine, and glucose. (Figure 5). However, in the loading column plot, some of the metabolites were shown to be not significant due to their error bars crossing the 0 axis of the plot. Consequently, these metabolites did not have a significant impact on the separation.

**Figure 6:**
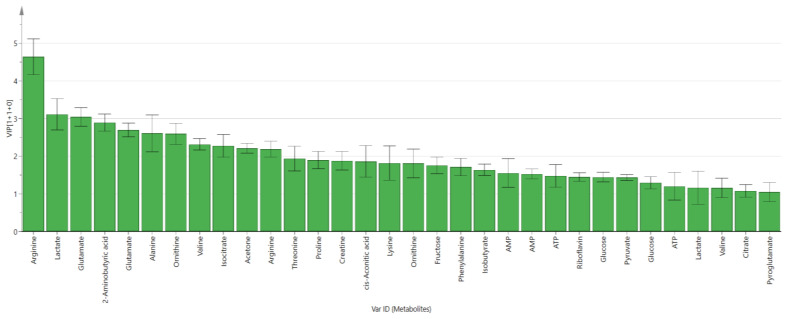
VIP values of the influential contributing metabolites in the OPLS-DA score plot of uninfected RBCs-control and uRBCs-CQ, error bars represent the standard error of the mean (SEM), uRBCs; uninfected red blood cells, CQ; chloroquine.

The VIP analysis of the second OPLS-DA model was conducted to identify the most important contributing variables, as shown in Figure 6. A total of 32 metabolites significantly contributed to the discrimination between the uRBCs treated with CQ and uRBCs-control with VIP values of more than 1. The metabolites were arginine, lactate, glutamate, 2-aminobutyric acid, glutamate, alanine, ornithine, valine, isocitrate, acetone, arginine, threonine, proline, creatine, cis-aconitic acid, lysine, ornithine, fructose, phenylalanine, isobutyrate, AMP, AMP, ATP, riboflavin, glucose, pyruvate, glucose, ATP, lactate, valine, citrate, and pyroglutamate. These metabolites had a significant impact on the separation and could be suggested as potential biomarkers.

### 2.4. The Model Validation of the OPLS-DA

**Figure 7:**
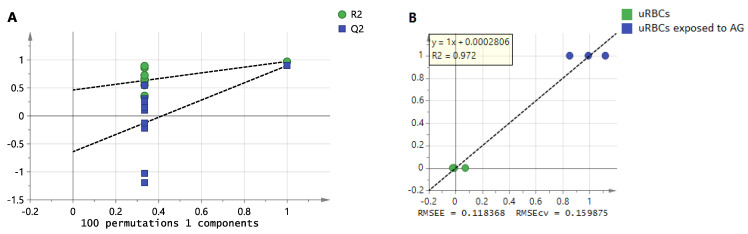
The OPLS-DA models’ validation of uRBCs-control and uRBCs-AG: (**A**) 100 permutations test; (**B**) the validation regression plot. uRBCs, uninfected red blood cells; AG, andrographolide.

To validate the OPLS-DA model, in the internal cross-validation, if the values of R2Y and Q2 are higher than 0.5, this indicates the model is valid [38]. R2 denotes the fitness of the model as well as describing the quality of Y variables through the model, while Q2 indicates the predictive diagnostics of the model. In the present study, by calculating R2Y and Q2, the goodness of fit and the predicting ability were determined. From the OPLS-DA model of uRBCs-AG and uRBCs, the model exhibited a high degree of fit, R2Y = 0.998, and a high degree of predictability, Q2 = 0.995. From the 100 permutations test, the model showed *Y*-axis intercepts of R2 less than 0.3 and those of Q2 less than 0.5 (R2 < 0.3, Q2 < 0.05), indicating that the models are valid and did not show overfitting [38]. The permutation tests with 100 permutations and external validation indicated that the OPLS-DA models were valid, as shown in Figure 7A.

Furthermore, this model is perceived to have excellent prediction and validation characteristics, which suggests that it is an adequate model. Additionally, for the established models, the validation for the various variables was verified using the regression diagnostics by the root mean square error of cross-validation (RMSECV) and root mean square error of calibration (RMSEC). Based on our results, the RMSEC and RMSECV were nearly similar, which implies that it is a good model (Figure 7B). These results suggest that each of these models meet the criteria of great validation and prediction performances.

**Figure 8:**
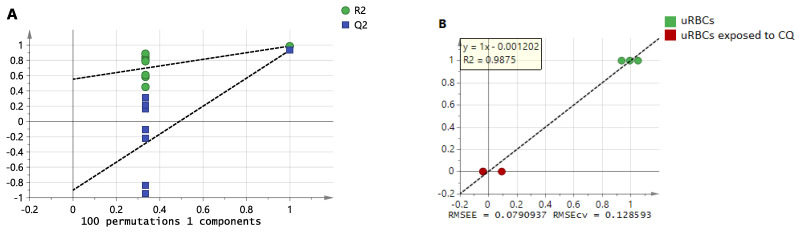
The OPLS-DA model’s validation of uRBCs-control and uRBCs-CQ: (**A**) 100 permutations test; (**B**) the validation regression plot. uRBCs, uninfected red blood cells; CQ, chloroquine.

The OPLS-DA model of uRBCs-CQ and uRBCs gave the results of R2Y = 0.903 and Q2 = 0.934, signifying that this model meets the criteria of the validation and prediction. In addition, the results of the 100 permutations test were acceptable, which indicates that the model is valid (Figure 8A). The regression coefficient (R^2^) was 0.987. Additionally, the difference between root mean square error of cross-validation (RMSECV) and root mean square error of calibration (RMSEC) was small. These findings show that the RMSEC and RMSECV are nearly similar, which means that it is a good model (Figure 8B). From these findings, it appears that this model has strong validity and prediction performances.

### 2.5. The Hierarchical Cluster Analysis (HCA)

**Figure 9:**
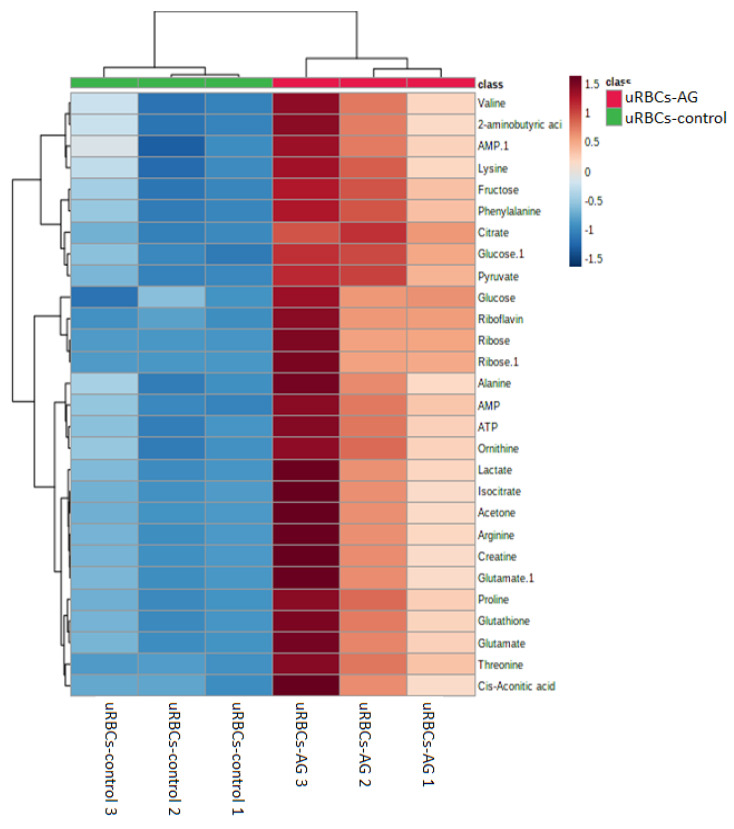
Heat map of the identified biomarkers of uRBCs and uRBCs-AG obtained from HCA by using Ward’s minimum variance method and Euclidean distance. The concentration of each metabolite is colored based on a normalized scale from minimum −1.5 (dark blue) to maximum 1.5 (dark brown). uRBCs, uninfected red blood cells; AG, andrographolide.

**Figure 10:**
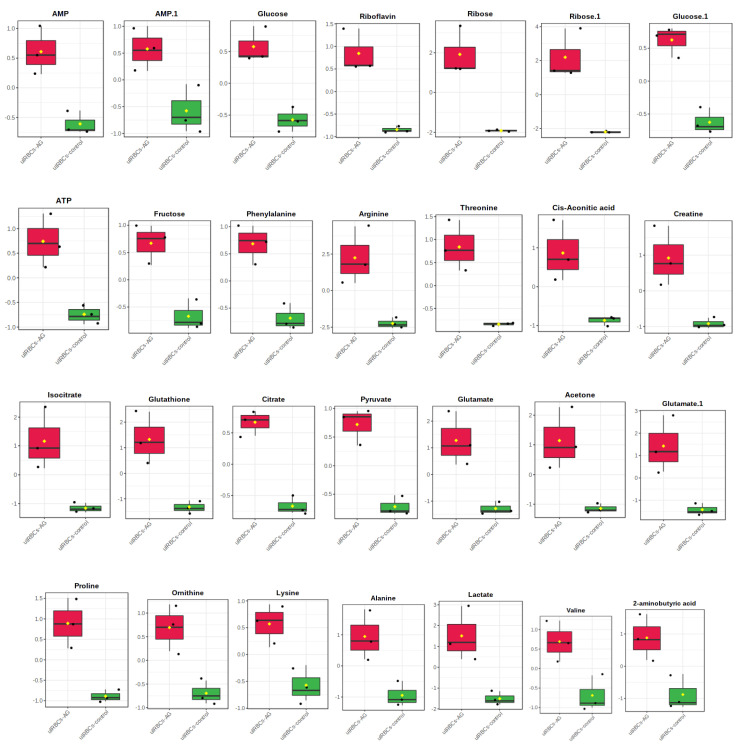
Box plots of the relative quantities of the significant metabolites of uRBCs-AG (red color) and uRBCs-control (green color) using ^1^H-NMR spectra binned data with a VIP value ≥0.1 in OPLS-DA model. uRBCs, uninfected red blood cells; AG, andrographolide.

A hierarchical clustering analysis (HCA) was conducted to deduce the metabolites’ variations in different groups of the uRBCs. Clustering analysis is dependent on the similitude concept. One method to identify the similarity between two objects in mathematical terms is the Euclidean distance. The samples were divided into two groups. The first HCA was between the uRBCs-AG versus uRBCs and the second one was for uRBCs-CQ versus uRBCs. Then the visual HCA was performed to identify the metabolites’ discrepancies for each group. From the results of the first of OPLS-DA plots and VIP plots of uRBCs-AG and uRBCs, the metabolites that contributed the most to the discrimination were determined. Before being exposed to HCA with Euclidean distance measurements and Ward’s clustering algorithm, the distinctive binned regions of the metabolites of importance in the group were normalized and Pareto-scaled. Figure 9 shows the results of the study as a HCA heat map, with each rectangle representing an averaged binned ^1^H-NMR spectral area indicative of the important metabolite, which was colored on a normalized scale from −1.5 (low) to 1.5 (high). The heat map successfully classified the group of uRBCs treated with AG as a large class that was different from uRBCs. In addition, the group of uRBCs treated with AG exhibited higher levels of arginine, ribose, lactate, glutamate, glutathione, isocitrate, acetone, alanine, creatine, 2-aminobutyric acid, proline, cis-aconitic acid, threonine, riboflavin, ATP, valine, pyruvate, ornithine, phenylalanine, fructose, citrate, AMP, and glucose. In contrast, these metabolites had lower levels in untreated uRBCs, as shown in Figure 9. The results suggest that in the levels of the metabolites in the treated uRBCs group, exposure to AG significantly contributed to the changes observed in many of the metabolites in the uRBCs [41]. In addition to that, it is possible that these results are due to the fact that AG has the effect of compromising RBCs’ membrane integrity [30]. As mentioned earlier, a total of 28 metabolites were identified as biomarkers in the group of uRBCs-AG. The relative quantification of these particular biomarkers was then assessed through binned data of average peak metabolites within the groups. The variations in the level of the metabolites were quantitatively evaluated as shown in Figure 10, which represents the box plots of these significant individual metabolites.

**Figure 11:**
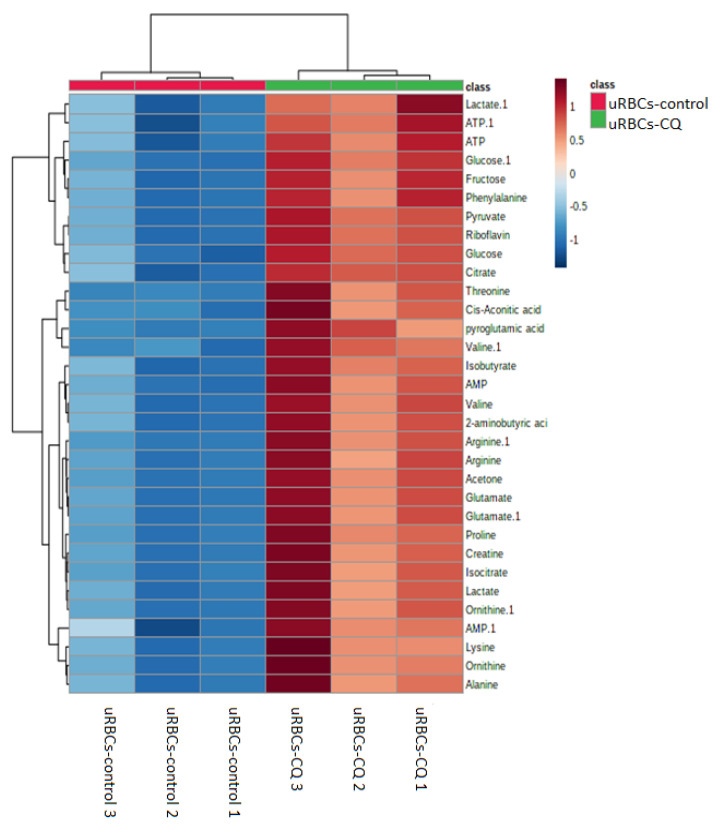
Heat map of the identified biomarkers of uRBCs-control and uRBCs-CQ obtained from HCA by using Ward’s minimum variance method and Euclidean distance. The concentration of each metabolite is colored based on a normalized scale from minimum −1 (dark blue) to maximum 1 (dark brown). uRBCs, uninfected red blood cells; CQ, chloroquine.

**Figure 12:**
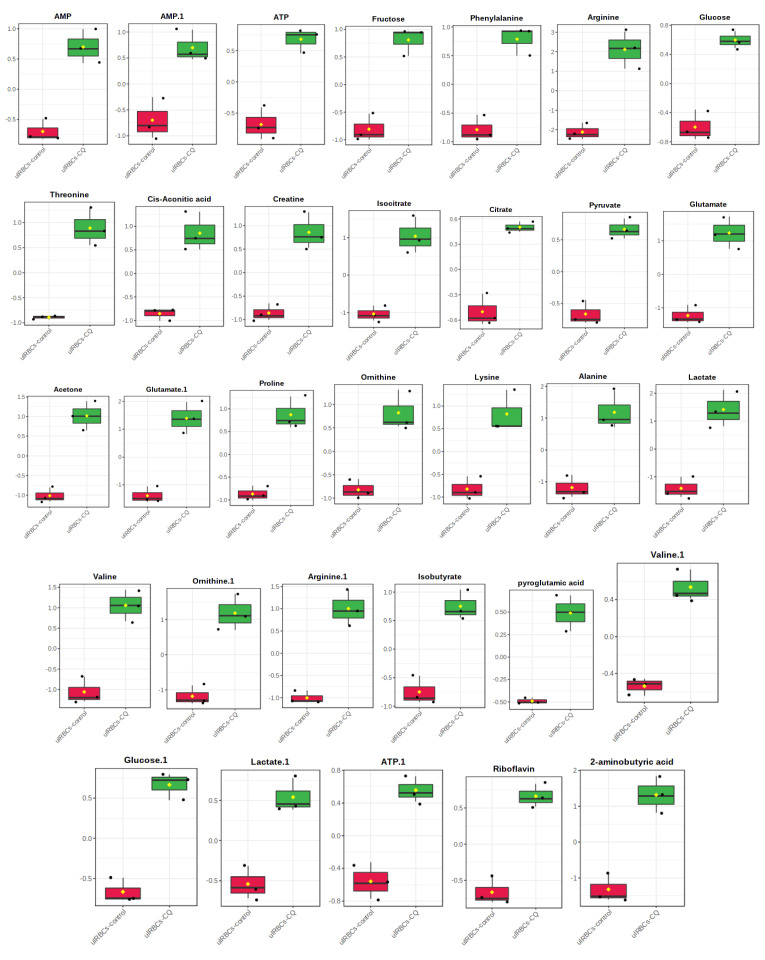
Box plots of the relative quantities of the significant metabolites of uRBCs-CQ (green color) and uRBCs (red color) using ^1^H-NMR spectra binned data with VIP value ≥0.1 in OPLS-DA model. uRBCs, uninfected red blood cells; CQ, chloroquine.

The other OPLS-DA plot and VIP plot of uRBCs-CQ and uRBCs in Figure 11 shows the results of the heat map analysis of identified biomarkers of uRBCs treated with CQ. The uRBCs group treated with CQ was categorized successfully as a broad group that differed from uRBCs. Furthermore, there were higher levels of the metabolites of uRBCs treated with CQ than in the uRBCs group. The metabolites were arginine, lactate, glutamate, 2-aminobutyric acid, glutamate, alanine, ornithine, valine, isocitrate, acetone, arginine, threonine, proline, creatine, cis-aconitic acid, lysine, ornithine, fructose, phenylalanine, isobutyrate, AMP, AMP, ATP, riboflavin, glucose, pyruvate, glucose, ATP, lactate, valine, citrate, and pyroglutamate (Figure 11). As previously described, a total of 32 metabolites in the uRBCs-CQ group were identified as biomarkers. The changes in metabolite levels were assessed via a comparison between uRBCs treated with CQ and the untreated group. Figure 12 shows the box plots of these important individual metabolites, relatively quantified to TSP.

### 2.6. Analysis of the Perturbed Metabolic Pathways by AG and CQ in Uninfected RBCs

**Figure 13:**
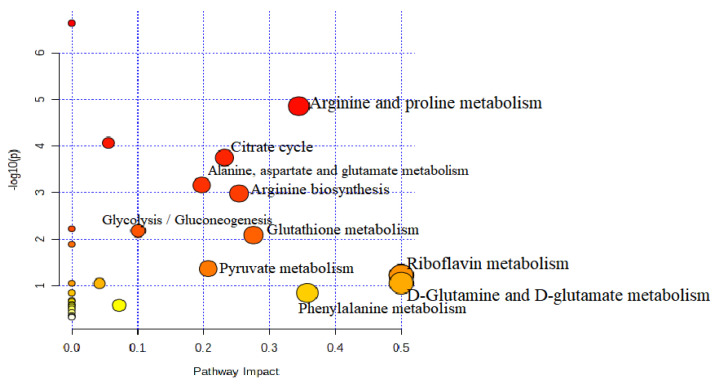
Summary of the pathway analysis of uninfected RBCs treated with AG.

The identified metabolite perturbations based on the ^1^H-NMR data are useful for extracting out the desired information from the uRBCs and uRBCs treated groups. To systematically identify the most significant pathways that are involved in these groups, metabolic pathway analysis (MetPA) using MetaboAnalyst (www.metaboanalyst.ca/MataboAnalyst) accessed on 20 March 2021 was performed as well as KEGG (https://www.genome.jp/kegg/pathway) accessed on 1 March 2021. The pathway impact factors are the appropriate tool to measure the significance of metabolites in the network. From the results, 28 metabolic pathways were identified in the group of uRBCs-AG (Table S1). Based on the set standards for a pathway impact value of higher than 0.1 [40] from the 28 metabolic pathways, 10 metabolic pathways were determined as important metabolic pathways in the uRBCs which were exposed to AG. The d-glutamate and d-glutamine metabolism as well as metabolic of riboflavin showed the same highest impact value of 0.50, followed by phenylalanine metabolism, arginine and proline metabolism, glutathione metabolism, arginine biosynthesis, citrate cycle, pyruvate metabolism, alanine, aspartate and glutamate metabolism, and glycolysis/gluconeogenesis with values of 0.35, 0.34, 0.27,0.25, 0.23, 0.20, 0.19, and 1.0, respectively (Table 2 and Figure 13).

**Figure 14:**
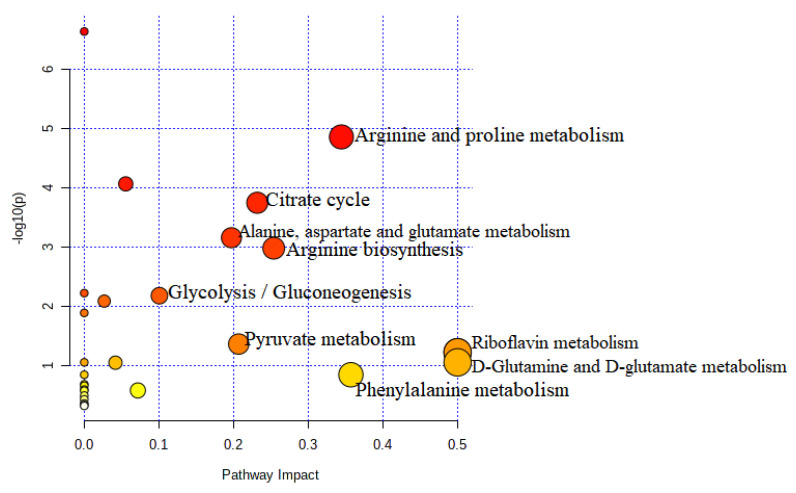
Summary of the pathway analysis of uninfected RBCs treated with CQ.

**Figure 15:**
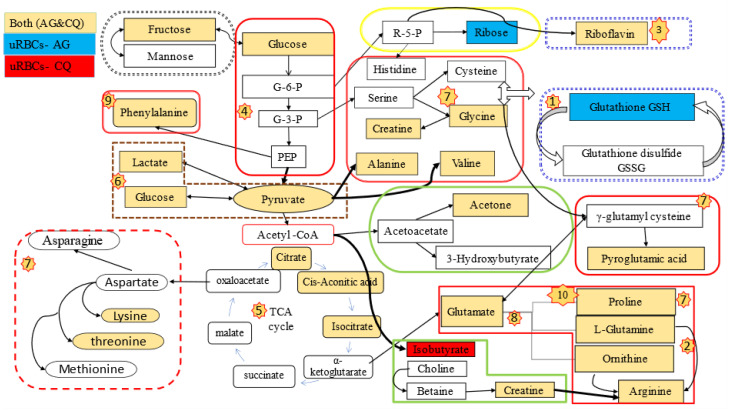
Schematic representation showing the perturbated metabolic pathways of the uninfected RBCs exposed to AG (uRBCs-AG) and the uninfected RBCs exposed to CQ (uRBCs-CQ) as identified by ^1^H-NMR. The metabolites that are outlined with light blue are present in uRBCs-AG, red are presents in uRBCs-CQ, whereas those highlighted in gold are present in both treated groups, uRBCs-CQ and uRBCs-AG. The metabolic pathways include, (1) glutathione metabolism; (2) arginine biosynthesis; (3) riboflavin metabolism; (4) glycolysis/c; (5) citrate cycle/TCA cycle; (6) pyruvate metabolism; (7) alanine, aspartate, and glutamate metabolism; (8) d-glutamine and d-glutamate metabolism; (9) phenylalanine metabolism; and (10) arginine and proline metabolism.

However, in the uRBCs-CQ group, the number of the identified metabolic pathways, including significant pathways, was less than in the uRBCs-AG group. Twenty-six metabolic pathways were identified in this group (Table S2). From 26 metabolic pathways, nine metabolic pathways were identified as important metabolic pathways in uRBCs treated with CQ. In addition, all affected metabolic pathways in this group were the same pathways identified in the uRBCs-AG group, except for glutathione metabolism, which was not significant because its impact value was less than 1.0. This is illustrated in Table 3 and Figure 14. Further, the general metabolic pathway map was constructed to illustrate all biomarkers and roles of each metabolite in the different pathways of the treated group of uRBCs-AG and uRBCs-CQ. All these metabolites took part in a number of pathways, which include amino acid, carbohydrate, and lipid metabolism. This schematic metabolic pathway is illustrated in Figure 15.

From the results of PAC and OPLS-DA, both treated groups of uRBCs-AG and uRBCs-CQ exhibited distinct variations in their metabolites compared to the uRBCs-control group. From the analysis of the perturbated metabolic pathways, ten metabolic pathways in uninfected RBCs treated with AG have been determined as disturbed. On the other hand, nine metabolic pathways in uRBCs treated with CQ were identified as disrupted metabolic pathways. In this section, the suggested metabolic pathways are further discussed in detail.

#### 2.6.1. Amino Acid Metabolism

In the human body, RBCs are the most abundant host cells. They represent a large proportion of the free amino acids (A.A.) in the blood [42,43,44], due to the fact that mature RBCs do not have nuclei, mitochondria, ribosomes, and other organelles and are unable to synthesize new proteins [4,5]. Nevertheless, various A.A transport systems have been identified in human RBCs, which permit the uptake of various exogenous biochemical compounds, such as amino acids, carbohydrates, and inorganic ions [45] or endogenous compounds, by the RBCs [34]. In addition, the transport system resembles those in other cells [46]. The results of the present study show an increase in the concentrations of most A.A. (such as arginine, alanine, threonine, valine, ornithine, glutamate, lysine, phenylalanine, methionine, glutamine, and proline) in the uRBCs exposed to CQ. The increased level of A.A. in this group suggests that the chloroquine might inhibit the RBCs in the uptake of some amino acids, thus increasing their levels [47]. Furthermore, the concentrations of the A.A. mentioned above were observed to rise in the uRBCs exposed to AG. The high levels of these A.A. in this group can be considered an early sign that the mechanism of action of AG is associated with levels of A.A. The results of HCA showed that there is an evident relationship between AG and CQ associated with a concentration of A.A. Therefore, it can be suggested that AG and CQ may have the effect of increasing the levels of A.A. of RBCs.

#### 2.6.2. Glutathione Metabolism

The current study results reveal a significant increase in the level of GSH in the uRBCs exposed to AG compared to the group of uRBCs exposed to CQ. Glutathione (GSH) is a tripeptide of low molecular weight, and it is well-known that GSH performs a crucial protective role versus oxidative processes, which damages the hemoglobin function and results in RBCs’ lysis [48,49]. In order to evaluate redox status, the antioxidants GSH and GSSG were previously determined and characterized by ^1^H-NMR in healthy RBCs [50,51,52]. The results of the current study reveal the differences in the levels of GSH in the treated groups, a result which is in alignment with Rossi et al.’s (2002) [53]. AG is one of the bioactive compounds of *A. paniculate* as a labdane diterpenoid derivative. Several publications have revealed the biological properties of AG, such as antimicrobial, anti-inflammation, and antioxidant [19,54]. The increase of GSH level can possibly be associated with the antioxidative property of AG. Moreover, these increases may be as a result of a conversion of GSSG to GSH [41]. It is known that the synthesis of GSH requires three amino acids, viz. glutamate, cysteine, and glycine, as well as two enzymes, glutamate-cysteine ligase (GCL) and GSH synthetase, which require ATP [48,55]. From the results, these A.A. were determined to be present at a high concentration, which in turn contributes to the increased synthesis of GSH.

#### 2.6.3. Carbohydrate Metabolism

The results showed high levels of glucose, ribose, and fructose in the uRBCs exposed to AG group compared to those in the uRBCs group. Meanwhile, the levels of glucose and fructose metabolites were high in the uRBCs exposed to CQ group. Such findings suggest that AG and CQ might affect the pathways associated with carbohydrate metabolism. The metabolism of carbohydrates is the most essential metabolic pathway, including glycolysis and gluconeogenesis, pyruvate metabolism and citrate cycle (TCA), due to its role in controlling and modulating the production of cellular energy in organisms. For example, the catabolism of glucose produces pyruvate with its derived ATP and NADH via the glycolysis pathway, which usually needs aerobic conditions for the conversion process to the acetyl-CoA essential for the TCA cycle [56]. In anaerobic conditions, glucose produces pyruvate, after which it is reduced to lactate [57]. As mentioned earlier, the metabolic pathways were identified as metabolic disturbances after exposure of the uRBCs to AG. These disorders in uRBCs may be attributed to AG (Table 2). Moreover, the results showed a notable increase in the level of lactate in both treated groups, uRBCs-CQ and uRBCs-AG, respectively, compared to the uRBCs group (Figure 9 and Figure 11). This is probably because of the increased lactate metabolism and the change in energy metabolism from glucose to lactate in the glycolysis pathway [57].

## 3. Materials and Methods

### 3.1. Chemicals and Consumables

**Figure 16:**
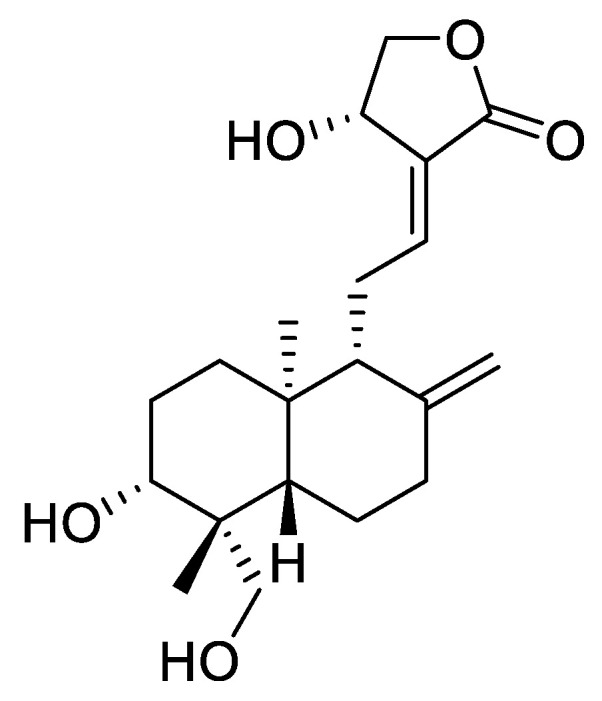
The chemical structure of the andrographolide compound.

Human O- erythrocytes, Roswell Park Memorial Institute medium (RPMI-1640) medium (1X) with sodium bicarbonate, Albumax II was procured from Gibco BRL (Grand Island, NY, USA). HEPES, triton X-100, EDTA, saponin, sorbitol, hypoxanthine, (100X) phosphate buffered saline (PBS), chloroquine diphosphate (CQ), NMR solvent: deuterium oxide 99.9% (D_2_O), trimethylsilyl-2,2,3,3-tetradeuteropropionate acid sodium salt (TSP), potassium hydroxide (KOH), and potassium dihydrogen phosphate (KH_2_PO_4_) were purchased from Sigma-Aldrich (St. Louis, MO, USA). Andrographolide compound C_20_H_30_O_5_ (Cat. No.: M16-11074), presented in Figure 16, was also obtained from Sigma-Aldrich. Gentamicin, sodium deuteroxide solution (NaOD), sodium chloride (NaCl) and hydro-chloric acid (HCl) were purchased from Jiangxi Dongxu Chemical Technology Co., Ltd, Kuala Lampur, Malaysia.

### 3.2. Blood Collection and Preparation for Culture

The first author donated uninfected RBCs (type O- negative) under the supervision of a hematologist. The blood was combined with citrate phosphate buffer as an anticoagulant (1:9 anticoagulant/blood). The blood was then washed three times with washing medium to remove plasma and white blood cells before being resuspended to generate an RBC suspension. In the washing media with RPMI-1640, the following combination was used, including 25 mM HEPES (4-(2-hydroxyethyl)-1-piperazine-ethan-sulphonic acid) buffer (pH 7.4), 24 mM sodium bicarbonate, 11 mM glucose, and 50 g/L gentamicin. The usual blood washing process was used as previously described [53].

### 3.3. In-Vitro Cell Culture of Uninfected RBCs

The uRBCs were cultured as described by Trager and Jensen [58] and suspended in complete RPMI-1640 malaria culture medium (cMCM) containing 25 mM HEPES, 0.75 mM hypoxanthin, Albumax 5%, 24 mM sodium bicarbonate, 11 mM glucose, and 20 µg/mL gentamicin at pH and hematocrit levels of 7.4 and 2%, respectively, in 75 cm^2^ tissue culture flasks. After that, all cultures were incubated at standard conditions for parasite cultivation in a micro-aerophilic environment containing 5% CO_2_ at 37 °C. The medium was changed daily. Before the drug exposure commenced, the uRBCs were established at 2% hematocrit. The first group was the untreated uRBCs cultures flasks which served as a control. The second group was uRBCs treated by exposure to AG at a concentration of 4.14 µM, which represented the IC_50_ of AG (the concentration of AG that inhibits malaria parasite growth at 50%), and the last group was uRBCs treated by exposure to CQ at a concentration of 20.19 nM (the concentration of CQ that inhibits malaria parasite growth at 50%), the IC_50_ of both AG and CQ were obtained from a previous study [59]. Next, the samples were collected from the control and uRBCs-CQ after 12 h of drug exposure. The uRBCs-AG were collected after 24 h; 12 and 24 h were the initial times of action of CQ and AG against the *P. falciparum* 3D7, which was determined in a previous study [59], respectively. For each group, five culture flasks were prepared, and the procedure was repeated three times.

### 3.4. The Extraction of Metabolites of Uninfected RBC

The uRBCs were extracted as described by Pertinhez et al. [16]. After 12 h and 24 h of drug exposure, the supernatant was removed from the cultures after centrifugation at 2000 rpm for 5 min at 4 °C. Then, the pellets of untreated uRBCs as well as treated uRBCs were individually washed in the 0.9% NaCl/10 mM phosphate buffer (pH 7). Following this procedure, the supernatant was discarded after centrifugation as stated earlier. This step was repeated twice. Subsequently, the samples were lysed through a cycle of freezing at −80 °C, thawed at 37 °C, vortexed for 20 s, and finally sonicated for 30 s. The procedure was repeated three times. Next, the proteins and membranes of all samples were excluded by ultrafiltration using a 5 kDa cut-off filter [60]. All the samples were centrifugated through a 5 kDa cutoff, and filtered at 14,000 rpm for 30 min at 4 °C [16]. Subsequently, 400 µL from each sample was transferred to an Eppendorf tube and mixed with 200 µL of phosphate buffer solution (KH_2_PO_4_) containing 0.1% trimethylsily l-2,2,3,3-tetradeuteropropionate acid sodium salt (TSP) in D_2_O (pH~7.4). A volume of 550 µL from each solution was transferred into a 5mm NMR tube. The NMR tubes were marked and subjected to ^1^H-NMR.

### 3.5. H-NMR Spectroscopy Analysis

All NMR spectra were acquired at 499.887 MHz on a 500 MHz Varian INOVA NMR spectrometer (Varian Inc., California, USA). The spectral analysis was carried out at room temperature with presaturation setting to suppress the residual water signal. Then, the transverse relaxation time of T2 measurement CPMG (Carr–Purcell–Meiboom–Gill) experiment was performed using the following parameters: (δ = 0.0002); (big δ = 0.4); a number of transient (n = 256 scans), and relaxation delay RD (D1 = 0.5 s). The CPMG spectrum was obtained in 16 min and 1 s. The CPMG experiment can reduce the broad signals of macromolecules and minimize the intensity to obtain a much better spectral baseline [61]. The TSP was used as the internal reference for the calibration of chemical shift at δ 0.0 ppm.

### 3.6. Processing Data of ^1^H-NMR Spectra and Metabolite Identification

One-dimensional (1D) ^1^H-NMR spectra were achieved on 500 MHz NMR spectrometer. All NMR spectra were processed by phased, baseline corrected, and calibrated to the internal standard (TSP) at 0.00 ppm. The NMR spectra were converted to ASCII files using Chenomx NMR suite software 7.5 (Chenomx Inc., Edmonton, Alberta, Canada). The NMR spectra were binned into 245 bins in the width of 0.04 ppm with the omission of the water region from (δ 4.75–4.90 ppm). MestReNova 6.0.2 software (Mestrelab Research, Santiago de Compostela, Spain) was used to process and assist for biomarker identification. The metabolites were identified by matching their signals with the library of Chenomx NMR database (version 7.5), recent publications on human blood and human serum metabolome [2,17], and the Human Metabolome Database (HMDB); http://www.hmdb.ca accessed on 14 March 2021 [62]. Afterwards, the NMR data were imported to the SIMCA-P software package (version 14.1) accessed on 14 March 2021. Pareto scaling was used to measure the data in order to reduce the effects of noise as well as make all metabolite signals the same strength [38].

### 3.7. Statistical Analysis

For the analysis and determination of variation between untreated and treated uRBCs, multivariate data analysis MVDA was used using the SIMCA-P software package (version 14.1). The data were analyzed and visualized by using an unsupervised statistical principal component analysis (PCA) and supervised statistical orthogonal partial least squares-discriminant analysis (OPLS-DA). In addition, the validation and significance of the model were carried out using the SIMCA-P software, 100 permutation tests, CV-ANOVA test, regression line, as well as the computation of R2Y and Q2Y values [38,40]. The heat map for the correlation analysis metabolites was carried out using MetaboAnalyst 4.0 (http://www.metaboanalyst.ca) accessed on 20 March 2021 [40]. The variable importance in projection (VIP) values of >1 [63] were used to determine the potential biomarkers in OPLS-DA. The pathway analyses were performed using the MetaboAnalyst 4.0 together with the Kyoto encyclopedia of genes and genomes (KEGG), and a database (http://www.genome.jp/kegg/ExPASy) accessed on 1 March 2021. The one-way analysis of variance (ANOVA) was performed using MetaboAnalyst. Tukey’s test was chosen as the post hoc analysis method and *p* ≤ 0.05 was considered to be statistically significant, while the values were expressed as mean ± SEM.

## 4. Conclusions

In conclusion, the experiments presented in this study were undertaken to determine the pharmacological effects of andrographolide as well as chloroquine on the metabolic variations of uninfected red blood cells (uRBCs) in vitro using a ^1^H-NMR-based metabolomics approach in combination with MVDA. Based on S-plot and VIP values, a total of 28 and 32 metabolites were identified as biomarkers in uRBCs-AG and uRBCs-CQ, respectively. In uRBCs treated with AG, ten metabolic pathways were determined as affected metabolic pathways, including riboflavin, d-glutamine and d-glutamate, phenylalanine, arginine and proline, glutathione, pyruvate alanine, aspartate, and glutamate metabolisms as well as arginine biosynthesis, citrate cycle, and glycolysis/gluconeogenesis. In contrast, in CQ-treated uRBCs, nine metabolic pathways were determined as disturbed metabolic pathways, which were the same disturbed metabolic pathways mentioned above for uRBCs-AG, except for glutathione metabolism. These findings suggest an evident relationship between AG and CQ associated with metabolic perturbations in intact RBCs after being exposed to the treatment. Our metabolomics results could allow valuable, comprehensive insights into the underlying mechanism of action of AG and CQ on red blood cells.

## Figures and Tables

**Table 1:** ^1^H-NMR characteristic signals of the identified metabolites in the red blood cells.

No.	HMDB ID	Metabolites	^1^H-NMR Signals
1.	HMDB0000718	Isovalerate	0.94 (d, 6.6), 1.93 (m)
2.	HMDB0000452	2-aminobutyric acid	0.98 (t, 7.6)
3.	HMDB0000883	Valine	1.02 (d, 7.1), 3.60 (d, 4.4)
4.	HMDB0001873	Isobutyrate	1.06 (d, 6.8)
5.	HMDB0000011	3-Hydroxybutyrate	1.20 (d, 6.2)
6.	HMDB0000190	Lactate	1.34 (d, 7.0)
7.	HMDB0000161	Alanine	1.48 (d, 7.3)
8.	HMDB0000182	Lysine	1.74 (m)
9.	HMDB0000214	Ornithine	3.06 (t, 7.5), 1.78 (m)
10.	HMDB0000162	Proline	1.99 (m), 3.33 (m), 2.06 (m), 4.13 (dd, 6.4, 8.7)
11.	HMDB0000719	Homoserine	2.02 (m)
12.	HMDB0000696	Methionine	2.14 (m)
13.	HMDB0001659	Acetone	2.22 (s)
14.	HMDB0000148	Glutamate	2.38 (m), 2.32 (m)
15.	HMDB0000243	Pyruvate	2.36 (s)
16.	HMDB0000641	l-Glutamine	2.44 (m)
17.	HMDB0000244	Riboflavin	2.49 (s), 2.56 (s)
18.	HMDB0000094	Citrate	2.66 (d, 15.1)
19.	HMDB0000125	Glutathione	2.18 (m), 2.58 (m), 2.98 (m)
20.	HMDB0000064	Creatine	3.02 (s)
21.	HMDB0000072	Cis-Aconitic acid	3.10 (s)
22.	HMDB0000097	Choline	3.22 (s)
23.	HMDB0003345	Glucose	5.22 (d, 3.8), 3.40 (m), 3.45 (m)
24.	HMDB0000123	Glycine	3.54 (s)
25.	HMDB0000167	Threonine	3.58 (d, 4.9)
26.	HMDB0000517	Arginine	3.78 (t, 6.5)
27.	HMDB0000043	Betaine	3.89 (s)
28.	HMDB0000283	Ribose	3.99 (m), 3.82 (m)
29.	HMDB0000169	Mannose	5.18 (d, 1.7), 3.86 (m)
30.	HMDB0000660	Fructose	4.01 (m)
31.	HMDB0000193	Isocitrate	2.95 (m)
32.	HMDB0000267	Pyroglutamic acid	2.50 (m), 4.16 (dd, 7.5, 5.0)
33.	HMDB0001890	N-Acetylcysteine	4.36 (m)
34.	HMDB0003391	2-Phosphoglycerate	4.50 (m)
35.	HMDB0001273	GTP	4.55 (d,5.0)
36.	HMDB0000017	4-Pyridoxate	4.74 (s)
37.	HMDB0000538	ATP	6.13 (d, 5.7)
38.	HMDB0000177	Histidine	7.06 (s)
39.	HMDB0000479	3-Methylhistidine	7.92 (s)
40.	HMDB0000045	AMP	8.23 (s), 8.58 (s)
41.	HMDB0000159	Phenylalanine	3.19 (m), 7.32 (d, 7.4)

The small letters in parentheses signify: s; singlet, d; doublet, dd; doublet of doublet, t; triplet, and m; multiplet. *J* couplings in Hz. HMDB; Human Metabolome Database, ATP; adenosine triphosphate, AMP; adenosine monophosphate, GTP; guanosine triphosphate.

**Table 2:** Results of the ingenuity pathway analysis of uninfected RBCs treated with AG by using MetaboAnalyst (MetPA) shown the impact values.

NO.	Pathway Name	Match	Raw *p*	-log(p)	Holm p	FDR	Impact
1.	Arginine and proline metabolism	6/38	0.0000141	4.8507	0.0011704	0.0005922	0.34441
2.	Citrate cycle (TCA cycle)	4/20	0.0001819	3.7401	0.014736	0.0038204	0.23173
3.	Alanine, aspartate and glutamate metabolism	4/28	0.0007073	3.1504	0.056585	0.011883	0.19712
4.	Arginine biosynthesis	3/14	0.0010628	2.9735	0.083964	0.01488	0.2538
5.	Glycolysis/Gluconeogenesis	3/26	0.0067171	2.1728	0.51721	0.070529	0.10065
6.	Glutathione metabolism	3/28	0.0082928	2.0813	0.63026	0.0774	0.27562
7.	Pyruvate metabolism	2/22	0.043961	1.3569	1.0	0.3357	0.20684
8.	Riboflavin metabolism	1/4	0.060569	1.2177	1.0	0.39137	0.5
9.	d-Glutamine and d-glutamate metabolism	1/6	0.089519	1.0481	1.0	0.47604	0.5
10.	Phenylalanine metabolism	1/10	0.14488	0.839	1.0	0.67609	0.35714

Raw *p*-values were defined according to a total number of hits and total compounds in each pathway; Holm p, *p*-value corrected by Holm–Bonferroni method, FDR, false discovery rate; impact, the pathway impact value computed from pathway topology analysis.

**Table 3:** Results of the ingenuity pathway analysis of uninfected RBCs treated with CQ by using MetaboAnalyst (MetPA) showing the impact values.

NO.	Pathway Name	Match	Raw *p*	-log(p)	Holm p	FDR	Impact
1.	Arginine and proline metabolism	6/38	0.000014	4.8507	0.001704	0.00059	0.34
2.	Citrate cycle (TCA cycle)	4/20	0.000181	3.7401	0.014736	0.00304	0.23
3.	Alanine, aspartate and glutamate metabolism	4/28	0.000707	3.1504	0.056585	0.0113	0.20
4.	Arginine biosynthesis	3/14	0.00128	2.9735	0.083964	0.018	0.25
5.	Glycolysis/Gluconeogenesis	3/26	0.00671	2.1728	0.51721	0.0729	0.10
6.	Pyruvate metabolism	2/22	0.043961	1.3569	1.0	0.335	0.21
7.	Riboflavin metabolism	1/4	0.060569	1.2177	1.0	0.39137	0.50
8.	d-Glutamine and d-glutamate metabolism	1/6	0.089519	1.0481	1.0	0.47604	0.50
9.	Phenylalanine metabolism	1/10	0.14488	0.839	1.0	0.67609	0.36

Raw *p*-values were defined according to a total number of hits and total compounds in each pathway. Holm p, *p*-value corrected by Holm–Bonferroni method, FDR, false discovery rate; impact, the pathway impact value computed from pathway topology analysis.

## Data Availability

The data used to support the findings of this study are included within the article and the Supplementary Information files. Any other data can be made available upon request.

## Supplementary Materials

The following are available online at https://www.mdpi.com/article/10.3390/metabo11080486/s1, Table S1: The ingenuity pathway analysis of uRBCs-AG by using MetaboAnalyst (MetPA), Table S2: The ingenuity pathway analysis of uRBCs-CQ using MetaboAnalyst (MetPA).
