# Supplementary material for: Investigation of Andrographolide Effect on Non-Infected Red Blood Cells Using the 1H-NMR-Based Metabolomics Approach
Source: Metabolites. 2021 Jul 28;11(8):486. doi: 10.3390/metabo11080486 (PMC8400355; doi:10.3390/metabo11080486)
Supplement: Supplementary file 1 [file metabolites-11-00486-s001.zip › metabolites-1272655-supplementary.pdf]

Table S1. The ingenuity pathway analysis of uRBCs-AG by using MetaboAnalyst (MetPA).

No.	Pathway Name	Match	p	-log(p)	Holm p	FDR	Impact
**1.**	Aminoacyl-tRNA biosynthesis	8/48	2.3528E-7	6.6284	1.9764E-5	1.9764E-5	0.0
**2.**	Arginine and proline metabolism	6/38	1.4101E-5	4.8507	0.0011704	5.9226E-4	0.34441
**3.**	Glyoxylate and dicarboxylate metabolism	5/32	8.7414E-5	4.0584	0.0071679	0.0024476	0.05556
**4.**	Citrate cycle (TCA cycle)	4/20	1.8192E-4	3.7401	0.014736	0.0038204	0.23173
**5.**	Alanine, aspartate and glutamate metabolism	4/28	7.0732E-4	3.1504	0.056585	0.011883	0.19712
**6.**	Arginine biosynthesis	3/14	0.0010628	2.9735	0.083964	0.01488	0.2538
**7.**	Valine, leucine and isoleucine biosynthesis	2/8	0.0060807	2.216	0.47429	0.070529	0.0
**8.**	Glycolysis / Gluconeogenesis	3/26	0.0067171	2.1728	0.51721	0.070529	0.10065
**9.**	Glutathione metabolism	3/28	0.0082928	2.0813	0.63026	0.0774	0.27562
**10.**	Glycine, serine and threonine metabolism	3/33	0.013126	1.8819	0.98446	0.11026	0.0
**11.**	Pyruvate metabolism	2/22	0.043961	1.3569	1.0	0.3357	0.20684
**12.**	Riboflavin metabolism	1/4	0.060569	1.2177	1.0	0.39137	0.5
**13.**	Nitrogen metabolism	1/6	0.089519	1.0481	1.0	0.47604	0.0
**14.**	D-Glutamine and D-glutamate metabolism	1/6	0.089519	1.0481	1.0	0.47604	0.5
**15.**	Cysteine and methionine metabolism	2/33	0.090675	1.0425	1.0	0.47604	0.04179
**16.**	Biotin metabolism	1/10	0.14488	0.839	1.0	0.67609	0.0
**17.**	Phenylalanine metabolism	1/10	0.14488	0.839	1.0	0.67609	0.35714
**18.**	Butanoate metabolism	1/15	0.20955	0.67872	1.0	0.92641	0.0

**19.**	Histidine metabolism	1/16	0.2219	0.65383	1.0	0.932	0.0
**20.**	Pantothenate and CoA biosynthesis	1/19	0.25788	0.58858	1.0	0.98431	0.0
**21.**	Purine metabolism	2/65	0.2665	0.57431	1.0	0.98431	0.07183
**22.**	Selenocompound metabolism	1/20	0.26951	0.56942	1.0	0.98431	0.0
**23.**	Pentose phosphate pathway	1/22	0.29226	0.53423	1.0	1.0	0.0
**24.**	Lysine degradation	1/25	0.32511	0.48797	1.0	1.0	0.0
**25.**	Porphyrin and chlorophyll metabolism	1/30	0.37663	0.42408	1.0	1.0	0.0
**26.**	Amino sugar and nucleotide sugar metabolism	1/37	0.44248	0.35411	1.0	1.0	0.0
**27.**	Valine, leucine and isoleucine degradation	1/40	0.46861	0.32919	1.0	1.0	0.0
**28.**	Tyrosine metabolism	1/42	0.48537	0.31393	1.0	1.0	0.0

Raw *p*-values were defined according to a total number of hits and total compounds in each pathway; Holm p, *p*-value corrected by Holm-Bonferroni method; FDR, False Discovery Rate; Impact, the pathway impact value computed from pathway topology analysis.

Table S2. The ingenuity pathway analysis of uRBCs-CQ by using MetaboAnalyst (MetPA).

No.	Pathway Name	Total	p	-log (p)	Holm p	FDR	Impact
**1.**	Aminoacyl-tRNA biosynthesis	8/48	2.3528E-7	6.6284	1.9764E-5	1.964E-5	0.00
**2.**	Arginine and proline metabolism	6/38	1.4101E-5	4.8507	0.0011704	5.926E-4	0.34
**3.**	Glyoxylate and dicarboxylate metabolism	5/32	8.7414E-5	4.0584	0.0071679	0.004476	0.06
**4.**	Citrate cycle (TCA cycle)	4/20	1.8192E-4	3.7401	0.014736	0.0038204	0.23
**5.**	Alanine, aspartate and glutamate metabolism	4/28	7.0732E-4	3.1504	0.056585	0.011883	0.20
**6.**	Arginine biosynthesis	3/14	0.0010628	2.9735	0.083964	0.01488	0.25
**7.**	Valine, leucine and isoleucine biosynthesis	2/8	0.0060807	2.216	0.47429	0.070529	0.00
**8.**	Glycolysis / Gluconeogenesis	3/26	0.0067171	2.1728	0.51721	0.070529	0.10
**9.**	Glutathione metabolism	3/28	0.0082928	2.0813	0.63026	0.0774	0.03
**10.**	Glycine, serine and threonine metabolism	3/33	0.013126	1.8819	0.98446	0.11026	0.00
**11.**	Pyruvate metabolism	2/22	0.043961	1.3569	1.0	0.3357	0.21
**12.**	Riboflavin metabolism	1/4	0.060569	1.2177	1.0	0.39137	0.50
**13.**	Nitrogen metabolism	1/6	0.060569	1.2177	1.0	0.39137	0.00
**14.**	D-Glutamine and D-glutamate metabolism	1/6	0.089519	1.0481	1.0	0.47604	0.50
**15.**	Cysteine and methionine metabolism	2/33	0.089519	1.0481	1.0	0.47604	0.04
**16.**	Biotin metabolism	1/10	0.090675	1.0425	1.0	0.47604	0.00
**17.**	Phenylalanine metabolism	1/10	0.14488	0.839	1.0	0.67609	0.36
**18.**	Butanoate metabolism	1/15	0.14488	0.839	1.0	0.67609	0.00

**19.**	Histidine metabolism	1/16	0.20955	0.67872	1.0	0.932	0.00
**20.**	Pantothenate and CoA biosynthesis	1/19	0.2219	0.65383	1.0	0.98431	0.00
**21.**	Purine metabolism	2/65	0.25788	0.58858	1.0	0.98431	0.07
**22.**	Selenocompound metabolism	1/20	0.2665	0.57431	1.0	0.98431	0.00
**23.**	Lysine degradation	1/25	0.26951	0.56942	1.0	1.0	0.00
**24.**	Porphyrin and chlorophyll metabolism	1/30	0.32511	0.48797	1.0	1.0	0.00
**25.**	Amino sugar and nucleotide sugar metabolism	1/37	0.37663	0.42408	1.0	1.0	0.00
**26.**	Valine, leucine and isoleucine degradation	**1**/40	0.44248	0.35411	1.0	1.0	0.00

Raw *p*-values were defined according to a total number of hits and total compounds in each pathway; Holm p, *p*-value corrected by Holm-Bonferroni method; FDR, False Discovery Rate; Impact, the pathway impact value computed from pathway topology analysis
